# Self-Efficacy and the Digit Ratio in a Group of Sports University Students

**DOI:** 10.3390/jfmk8030097

**Published:** 2023-07-13

**Authors:** Simona Massimino, Sergio Rinella, Claudia Savia Guerrera, Donatella Di Corrado, Romina Astuto, Alessia Sorbello, Francesca Reina, Vincenzo Perciavalle, Andrea Buscemi, Marinella Coco

**Affiliations:** 1 Department of Biomedical and Biotechnological Sciences, Section of Physiology, University of Catania, 95100 Catania, Italy; 2 Department of Educational Sciences, University of Catania, 95100 Catania, Italy; 3 Department of Sport Sciences, Kore University, 94100 Enna, Italy; 4 Faculty of Medicine, Kore University, 94100 Enna, Italy; 5 Study Center of Italian Osteopathy and Horus Social Cooperative, 95100 Catania, Italy

**Keywords:** emotion control, personality, state–trait anxiety, testosterone, gender differences

## Abstract

Self-efficacy is one’s awareness of being able to cope optimally with different situations. Perceived self-efficacy is a belief that closely involves emotional and personological functioning. In fact, when one perceives oneself as capable, the likelihood of success increases significantly. The aim of this research was to verify a possible correlation between self-efficacy and the Digit Ratio (2D:4D), referred to as prenatal androgen levels, and whether these correlate with some psychological variables, considering possible gender differences. This study involved 56 sports university students, whose 2D:4D ratio was calculated. Moreover, self-assessment questionnaires: the Self-efficacy Scale (SES), the Big Five Questionnaire-2 (BFQ-2), the Profile of Mood State (POMS), the State–Trait Anxiety Inventory Form Y (STAI-Y) and the Emotional Regulation Questionnaire (ERQ) were administered. The data show that the perceived levels of self-efficacy seem to be influenced by an innate predisposition linked to prenatal androgen values to which the subjects were exposed during uterine life (2D:4D). This negative correlation indicates that the higher the value of the digit ratio, the more likely it is that the level of perceived self-efficacy will be lower. Finally, the degree of perceived self-efficacy also seems to depend on the levels of subjective anxiety, understood both as a personological disposition and a contingent condition, and the latter also seems to be influenced by prenatal androgen levels, particularly in women.

## 1. Introduction

Self-efficacy is the belief that individuals have that they are able to cope optimally in different situations. It is about perceiving oneself as able to manage the difficulties that may occur during task performance [1,2], and it also correlates with self-assertion.

In fact, self-efficacy is not a personality trait, but it is deeply linked with it: perceiving oneself as being able to face trials and actually achieve objectives is motivating and satisfying, and consequently, this belief will influence how and what one learns; this aspect will have an important role in the individual’s long-term evolution [3,4].

The more capable you feel, the more likely you are to experience success by employing contextually appropriate resources. Conversely, the less capable you feel, the less likely you are to achieve your goals. Repeated flops will undermine the individual’s motivation and self-esteem.

Around the end of the 1990s, an experimental study allowed us to confirm the above: a sample of subjects was assigned a task, and the ones who were convinced to have the skills to solve it showed greater commitment, used more resources, and consequently, found better solutions [5].

More recent studies confirm that high levels of self-efficacy correlate with good task success [6], correlate with lower levels of stress and anxiety [7], and impact psychological well-being and personal achievement [8,9,10].

Perceived self-efficacy is clearly a belief that is strongly related to emotional functioning. Emotions play a leading role in any moment of our lives and influence both the way we experience events and the way we interact with others [11,12,13].

Today we discuss emotional intelligence as a skill appropriately defined, which refers to the ability of the subject to understand their emotions in terms of self-awareness and also to be able to describe and understand emotional states in an appropriate way according to different contexts. Emotional intelligence finds its greatest importance in the ability to know how to use emotion regulation strategies to cope with emotionally intense situations [14]. The most commonly used strategies are cognitive reappraisal and emotional suppression: the first acts in an adaptive direction, correctly redirecting the emotional reaction triggered; the second is activated through the suppression of emotion and has an inhibitory action, which often becomes counterproductive [15]. Modern neuroscience has coined the term affective neuroscience, referring to the branch of this discipline dedicated to the study of emotions, which has increased knowledge about brain mechanisms and physiological and anthropometric measurements to “localize emotional states” [16]. In the study of human functioning, recent research [17,18] has revealed the influence on affective states and psychological variables of the digit ratio, denoted by the formula 2D:4D, which refers to the ratio of the length of the second to the fourth finger of the hand, usually the right, which is strongly dependent on the level of testosterone absorbed by the fetus during intrauterine life; specifically, this index differs for males and females because the concentration of androgen receptors in the fourth finger of the hand influences the development of its length. Therefore, usually, this value is lower in men as they have a greater fourth finger length derived from a higher concentration of androgen receptors, whereas this value is higher in women as a result of a lower concentration of androgen receptors resulting in a shorter fourth finger length [19].

As it is known, the level of prenatal hormones has an influence on adult brain organization [20] and also on the behavior that is manifested [21,22]. From this, it is possible to identify, in particular, a correlation between personality and testosterone levels. Early studies on this topic, some of which were conducted with prisoners and psychiatric patients, verified an association between high testosterone levels and aggressive behavior [23,24]. The same correlations have been observed in populations of healthy, non-deviant subjects [25]. More research has investigated and confirmed this correlation: high plasma levels of testosterone are associated with personality traits such as hostility, sensation-seeking, risk-taking or dominance in males [26,27]. However, regarding research on females, this has not clarified the influence of testosterone on aggressive and antisocial behavior [28]. Lower 2D:4D ratio levels are found to correlate with greater physical aggression in males, a distinction that does not occur for females [29].

Peter Eachus [30] investigated in a study a possible correlation between digit ratio and Internet self-efficacy. On this construct, previously measured by the Web User Self-Efficacy (WUSE) scale, gender differences were found overall, with men having higher Internet Self-Efficacy. It was therefore hypothesized that there might be a statistically significant correlation between self-efficacy measures and the digit ratio.

However, another study by Wade et al. [31] focused on a female sample, investigating and confirming the above. In particular, the authors in this study aimed to investigate the correlation between perceived self-efficacy, body esteem, personality and digit ratio.

Evardone and Alexander [32] investigated the relationship between 2D:4D and anxiety, finding a positive correlation between the value of the digit ratio and anxiety levels in men, i.e., men with higher levels of 2D:4D have higher levels of anxiety; according to another study, high levels of the 2D:4D ratio also correlate with higher rates of depression in men [33]; in addition, it seems that a low 2D:4D ratio is a protective factor against anxiety in both men and women, and this protection appears to act throughout life [34].

In order to have the correct emotional response in different situations, it is important to be able to regulate one’s emotions, which influence one’s perceived self-efficacy. From this assumption comes our interest in investigating the variables that most correlate with it in order to understand if the use of regulation strategies is in some way dependent on other characteristics that the subject can or cannot possess.

The purpose of our study was to investigate whether and how physiological characteristics, such as testosterone levels, could affect psychological characteristics such as Self-efficacy; in addition, we assessed other affective states and psychological variables that may influence self-efficacy, such as anxiety, personality and emotional intelligence; and finally, we aimed to evaluate if there were any gender differences in our results.

## 2. Materials and Methods

### 2.1. Participants

The research was conducted on university students of the Master’s degree course in Motor Sciences at the University of Catania; out of 100 students enrolled in the course, only 56 joined.

Fifty-six young sports and healthy volunteers, aged between 21 and 31 years, had a mean (M) age of 25.78 years with a standard deviation (SD) of ±2.66; of these, 32 were female (M = 25.53 ± 2.99 SD) and 24 were male (M = 26.12 ± 2.15 SD). Table 1 shows the descriptive characteristics of the sample, divided by gender (Table 1A female sample, Table 1B male sample) and age.

Specifically, for this study, we chose a group of healthy and physically performing college students in order to minimize any occurrence of physical illnesses or psychological syndromes, e.g., body dysmorphic disorder, that might have influenced the collected data. Moreover, participants excluded from this study were those who obtained a standardized score (T-score) ≥ 65 on the Lie Scale and at least on 3 major factors of the BFQ-2. The authors of the BFQ-2 indicate this criterion to identify the falsified personological profiles that should be excluded from data analysis. All participants provided written consent and were informed about procedures and their rights to anonymity, in accordance with the Declaration of Helsinki.

### 2.2. Measures

To carry out this study, our research protocol required that the subject first complete an anamnestic grid that contained general information. Then, they were administered the Big Five Questionnaire 2 (BFQ-2) to investigate their personality; the Digit ratio (2D:4D) was used as an indirect measure of pre-natal androgen levels during intrauterine life; the Profile of Mood States (POMS) provided a measure of their mood states; the STAI-2 (State–Trait Anxiety Inventory) measured their anxiety; in order to evaluate their self-efficacy, the Self-efficacy Scale was administered; and finally, to assess their emotional intelligence, the ERQ (Emotional Regulation Questionnaire) was used. All instruments are detailed below:

Digit ratio: For the measurement of the 2D:4D ratio, direct measurements were made of the length of the second and fourth fingers of the right hand for males and of both hands for females, following the methodology already found in the literature [17,35]; the length was measured twice, by having the palm of the open hand resting on a plane and using a meter to obtain the value of the length that goes from the metacarpophalangeal fold to the fingertips. This fold, which constitutes one of the fundamental folds of the hand, is believed to form around the 9th week of the gestational period [36]. The 2D:4D ratio has been reported only in reference to the right hand, as the ratios between the fingers of this hand appear more sensitive to prenatal androgens [37].

BFQ-2 (Big Five Questionnaire 2): The Italian version developed by Caprara et al. [38] was used to measure personality traits related to Costa and McCrae’s 1990 theory (Big Five Theory). It is a self-report questionnaire consisting of 134 Items that subjects must answer using a 5-point Likert scale, ranging from “Absolutely false to me” to “Absolutely true to me”. Results are summed across dimensions and sub-dimensions to then identify the 5 factors, each divided into two subscales, as follows:

This instrument examines the five personality factors, each divided into two subscales, as follows:

(1) Energy refers to the concept of extroversion in the original model and determines the activity and dynamism of a subject (Cronbach’s alpha = 0.81). The subscales of reference are Dynamism, understood in terms of energy and activity, and Dominance, which refers to the tendency to excel and dominate over others.
(2) Agreeableness refers to the concept of agreeableness in the original model and refers to a subject’s altruism, sociability, and empathy toward others (Cronbach’s alpha = 0.73); the two subscales are Cooperativeness/Empathy, and Politeness/Friendliness.
(3) Conscientiousness refers to the capacity for self-control and determination, and therefore the higher it is, the more it refers to precise, orderly, persevering subjects (Cronbach’s alpha = 0.81); the subscales refer to Scrupulousness and Perseverance, the first referring to order and reliability, the second to persistence and pursuit of objectives.
(4) Emotional Stability refers to the attitude of the subject in the self-regulation of their own emotional states, and higher scores indicate low levels of anxiety, stress, vulnerability, irritability, etc.; the two sub-dimensions measure the Control of Emotions, or the emotional states that the subject experiences, and the Control of Impulses, which evaluates the self-control of these in particular situations of discomfort and stress.
(5) Openness refers to culture, interest in events that occur in the world and new experiences (Cronbach’s alpha = 0.75); the subscales are Openness to culture and Openness to experience: first refers to interest in reading and knowledge, second to the predisposition towards new situations and challenges, and third to openness to lifestyles different from one’s own.

To these factors is added the measurement of a sixth scale, called Lie, relating to social desirability, which is also divided into two subscales, one egoistic and the other moralistic. The first refers to the tendency to attribute high intellectual abilities to oneself. The second refers to morally correct behavior and to the tendency to describe oneself as an affable person (Cronbach’s alpha = 0.74) [38]. This scale, not only provides a measure of the degree of social desirability shown by the subject but also has the main function of identifying attempts to falsify answers and, therefore, provides a measure of reliability.

The scores for each of the five traits, together with the Lie scale, range from very low (between 25 and 35), low (between 36 and 45), normal (between 46 and 55), high (between 56 and 65) and very high (between 66 and 75); the final raw scores are corrected for an average reference sample by gender and age.

STAI-Y (State–Trait Anxiety Inventory Form Y) [39]: The test consists of 40 items and is divided into two parts: the first 20 items assess state anxiety, referring to the psychophysiological condition of a given moment, while the other 20 items measure trait anxiety, understood as a personality trait; the subject expresses a value for each statement with a score ranging from 1 (not at all) to 4 (very much), with a total score ranging from 20 to 80 for both parts: the higher the score, the greater the reference anxiety state [40]. The resulting score is then adjusted for the gender and age of the subject. Internal consistency coefficients for the scale have ranged from 0.86 to 0.95, and test-retest reliability coefficients have ranged from 0.65 to 0.75 over a 2-month interval.

GSE (General Self-efficacy Scale) [41]: It is a scale built on samples from 23 nations (the scale is available in 33 languages), with Cronbach’s alphas ranging from 0.75 to 0.90, with the majority ranging over 0.80. It consists of 10 items that assess the subject’s general self-efficacy, with a reference value for each item ranging from “Not at all true” to “Totally true”. The higher the score, the higher the perceived self-efficacy of each subject.

POMS (The Profile of Mood States) [42]: This instrument is composed of 58 different adjectives, referring to as many moods that the subject has experienced in the last week and that he describes on a Likert scale ranging from 0 (not at all) to 4 (very much). The items are combined in such a way as to provide value relative to six different factors: Tension (T; Cronbach’s alpha = 0.76), Depression (D; Cronbach’s alpha = 0.66), Anger (A; Cronbach’s alpha = 0.89), Vigor/activity (V; Cronbach’s alpha = 0.87), Fatigue (F; Cronbach’s alpha = 0.95) and Confusion (C; Cronbach’s alpha = 0.70). In addition, it provides a global index of mood state called TMD (Total Mood Disturbance), which is obtained by summing the values of all factors except for the V factor, which is subtracted.

ERQ (Emotional Regulation Questionnaire) [43]: The instrument measures two emotion regulation strategies: cognitive reappraisal and emotional suppression; the first concerns the ability to modify the emotional response to an emotionally susceptible event before it occurs, while the second inhibits the emotional response produced so that it is not expressed. It consists of 10 items for each of the two strategies, both related to negative and positive emotions and involving two aspects: emotional expression and emotional experience. The subject has to assign each item a value on a Likert scale ranging from 1 to 7, that is, from ‘strongly disagree’ to ‘strongly agree’. Higher scores correspond to a greater use of this regulation strategy. Cronbach’s α reliability coefficients are 0.84 for the Reappraisal scale and 0.72 for the Suppression scale; item-total correlations ranged from 0.48 to 0.68 for Reappraisal and from 0.42 to 0.63 for Suppression.

In this study, we used established self-assessment methods, such as POMS and ERQ. The choice is mainly due to the simplicity of their administration and the ease of understanding by the participants. However, self-assessment measures, although widely used, can be susceptible to bias and may not fully capture actual self-efficacy. To reduce this risk, methods have been proposed that use physiological tools for analyzing participants’ perceptions, such as Dang and coworkers [44], Castiblanco Jimenez and coworkers [45] and Shi [46], which, however, are less easy to administer and whose results are slower to collect.

### 2.3. Data Analysis

Data were collected, averaged, and then compared with the paired t test (two-tailed). Moreover, linear regression and the correlation coefficient of Pearson were also calculated. Significance was set at *p* < 0.05, and all data are reported as mean ± standard deviation (SD). Statistical evaluation was carried out using SPSS 23.0 (SPSS Inc., Chicago, IL, USA).

## 3. Results

The measurement of 2D:4D ratios was calculated in centimeters (cm) and resulted in mean values of 0.99 ± 0.04 cm. The difference in 2D:4D ratios between males (0.99 ± 0.04 cm) and females (1.00 ± 0.04 cm) was not statistically significant. Means and standard deviations relative to the scores obtained in the ERQ, separated into the two subscales of the test (Cognitive Reappraisal and Emotional Suppression), are reported in Table 2. As shown, subjects tended to report higher scores in cognitive reappraisal than in emotional suppression, both in the whole sample and separately in the male and female samples.

Table 3 and Table 4 show the means and standard deviations obtained at the STAI-Y and POMS tests.

Table 5 shows the means and standard deviations of the five personality factors obtained from the administration of the Big Five Questionnaire-2.

In the total sample, the personality trait that obtained the highest average score is Energy, while the lowest score reported is related to Emotional Stability, which represents the lowest score in both males and females. Finally, the trait of Agreeableness has the highest mean score in the female sample, while Openness has the highest mean score in the male sample. Lastly, the mean values obtained in Self-efficacy are 19.66 ± 5.47 SD (males: 21.38 ± 4.97; females: 18.38 ± 5.55).

### 3.1. Digit Ratio (2D:4D)

**Figure 1:**
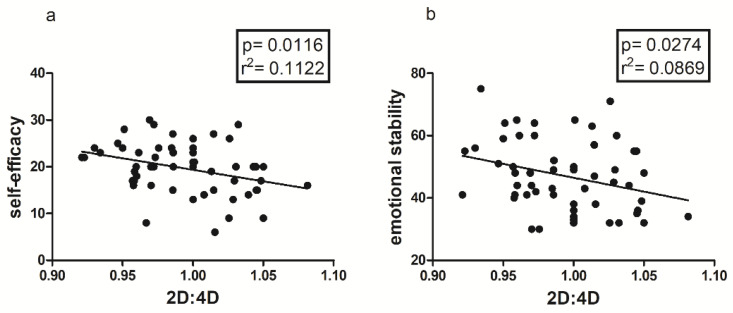
Correlation between digit ratio (2D:4D), (**a**) Self-efficacy and (**b**) Emotional Stability (BFQ-2) in the entire sample.

From the results obtained from the 2D:4D ratio analysis, in the total sample, no statistically significant correlation is observed with Emotional Intelligence, while there is a negative correlation with Self-efficacy (*p* = 0.0116; r^2^ = 0.1122), the personality trait Emotional Stability (*p* = 0.0274; r^2^ = 0.0869), and its subscale Emotional Control (*p* = 0.0311; r^2^ = 0.0832), as shown in Figure 1.

**Figure 2:**
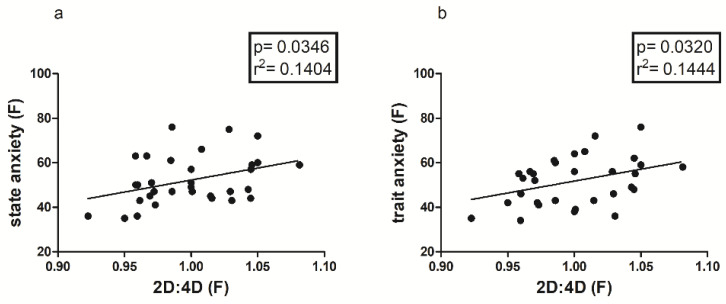
Correlation between digit ratio (2D:4D) and both State (**a**) and Trait Anxiety (**b**) of STAI-Y in the female sample.

Concerning the relation with the STAI-Y, the 2D:4D correlates positively only in the female sample with both State (*p* = 0.0346; r^2^ = 0.1404) and Trait Anxiety (*p* = 0.0320; r^2^ = 0.1444), as shown in Figure 2. In relation to personality traits, only a negative correlation is reported with the Cooperativeness subscale (*p* = 0.0141; r^2^ = 0.1848).

### 3.2. Self-Efficacy and Other Psychological Variables

**Figure 3:**
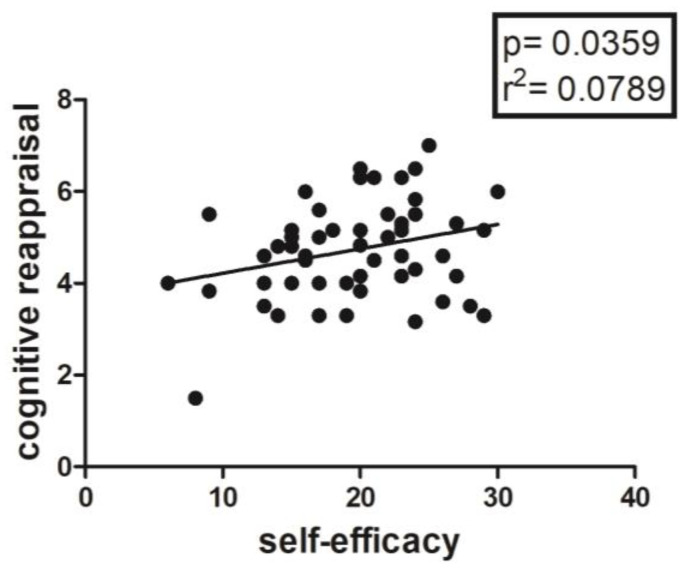
Correlation between Self-Efficacy and Cognitive Reappraisal (ERQ) in the entire sample.

Regarding ERQ, specifically, Cognitive Reappraisal in the total sample shows a positive correlation with Self-efficacy (*p* = 0.0359; r^2^ = 0.0789), as can be seen in Figure 3. Moreover, a positive correlation emerged between Suppression and Trait Anxiety (*p* = 0.0044; r^2^ = 0.1405) and a negative correlation with Agreeableness (*p* = 0.0038; r^2^ = 0.1449) and its subscales, Cooperativeness (*p* = 0.0073; r^2^ = 0.1260) and Friendliness (*p* = 0.0170; r^2^ = 0.1009). In addition, a negative correlation emerged between Suppression and the Dynamism subscale (*p* = 0.0174; r^2^ = 0.1003).

**Figure 4:**
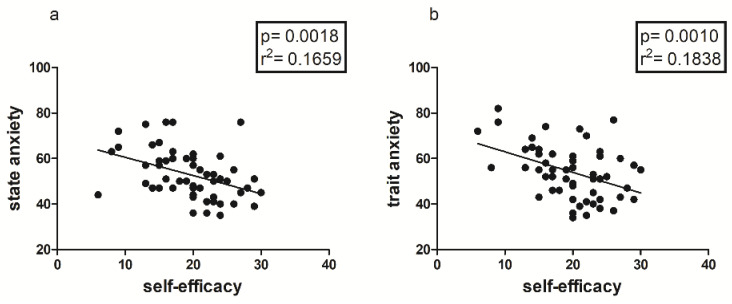
Correlation between Self-efficacy and both State (**a**) and Trait Anxiety (**b**) of STAI-Y in the entire sample.

**Figure 5:**
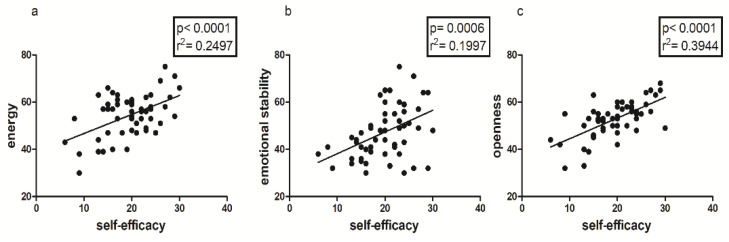
Correlation between Self-efficacy and Energy (**a**), Emotional Stability (**b**) and Openness (**c**) of BGQ-2 in the entire sample.

As shown in Figure 4 and Figure 5, Self-efficacy showed statistically significant correlations with both State and Trait Anxiety (Figure 4) and with some Big Factors of BFQ (Figure 5) in the total sample. In addition, Self-efficacy showed a positive correlation with Vigor (*p* =< 0.0001; r^2^ = 0.2594) and a negative correlation with TMD (*p* = 0.0385; r^2^ = 0.0769), factors of the POMS.

**Figure 6:**
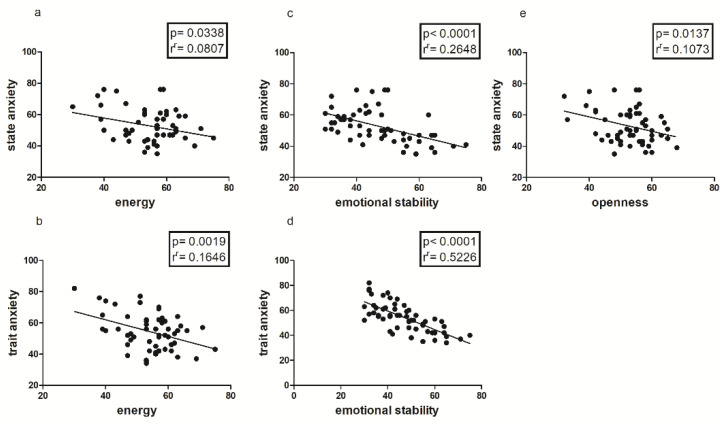
Correlations between State–Trait Anxiety (STAI-Y), Energy (**a**,**b**), Emotional Stability (**c**,**d**) and Openness (**e**) of BFQ-2 in the entire sample.

In addition, both state and trait anxiety show positive correlations with all factors of the POMS (*p* =< 0.001), except for Vigor, with which it correlates negatively. State and Trait anxiety also show negative correlations with most of the BFQ-2 Big Factors, as shown in Figure 6.

### 3.3. Gender Differences

**Figure 7:**
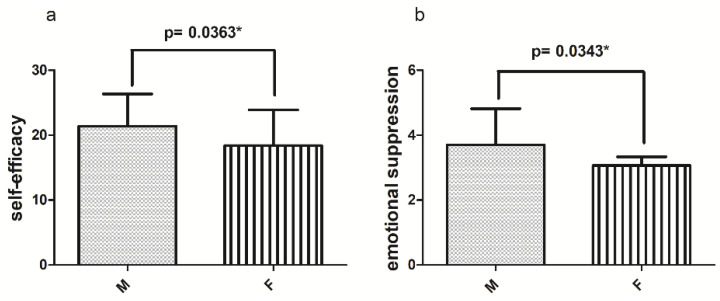
On the left are the mean values of Self-efficacy (**a**) and on the right are the mean values of Emotional Suppression (ERQ) (**b**), both in male and female samples; * *p* < 0.05, statistically significant difference.

Comparison of the results obtained from the male and female samples separately, as shown in Figure 7, revealed a statistically significant difference in the mean values reported on the Self-Efficacy Scale, where the male sample obtained statistically higher mean values (21.38 ± 4.97) than the female sample (18.38 ± 5.55). In addition, again, the male sample, when comparing data from the ERQ, tended to show statistically significant higher mean Emotional Suppression values (3.67 ± 1.11), compared to women (3.06 ± 1.53).

## 4. Discussion

The purpose of our research was based on the hypothesis of a correlation between perceived self-efficacy and prenatal androgen exposure levels, as expressed by the 2D:4D ratio. The analysis of the results revealed a negative correlation between self-efficacy and the 2D:4D ratio, suggesting that the perception of one’s self-efficacy appears to be somewhat influenced by an innate predisposition related to the prenatal androgen values to which individuals were exposed during uterine life; therefore, it would seem that the higher these values are (i.e., a lower level of prenatal androgen exposure), the lower the level of perceived self-efficacy will be. In addition, a statistically significant difference between male and female values emerged from the results, and what was discussed could provide an explanation: as can be seen from the graph (Figure 7a), a higher mean value of self-efficacy was found in the male sample, which differs significantly from the female sample; therefore, if what was found by the correlation between the 2D:4D ratio and self-efficacy is true, this could be partly explained by the fact that males normally have a higher level of prenatal androgens [47] and therefore males might have an innate predisposition to higher levels of Self-efficacy.

Another important finding is represented by the positive correlation between 2D:4D ratio and State and Trait Anxiety, only in the female sample (Figure 2); this could mean that there is an innate predisposition to anxiety both as a contingent physiological condition and as a personality trait, which seems to be correlated with prenatal androgen levels [48,49], and in particular with lower androgen levels [34]. As is well known, anxiety and personal emotional condition in general are determinants of perceived levels of self-efficacy, which seem to be partly conditioned by predetermined factors, such as the 2D:4D ratio [17,18], as confirmed in our study by both the negative correlation between anxiety and self-efficacy (Figure 3, Figure 4 and Figure 5b) and the positive correlation between the 2D:4D ratio and emotional stability (Figure 1).

Limitations. It should be noted that, although in the literature there are many studies that associate the 2D:4D ratio with a whole series of variables (such as risk-taking, aggressive behavior, etc.), other studies have failed to reproduce these associations. Recently, James M. Smoliga and colleagues [50] published a paper analyzing possible sources of error in studies involving the 2D:4D ratio. They conclude by listing four possible “pitfalls” that could avoid unreliable results, namely: (1) pre-registration of the study protocol; (2) performing or reporting a priori power analysis; (3) detailed accounting for multiple comparisons; and (4) reporting negative findings from other outcomes. In setting up the present study, we tried to apply these suggestions.

## 5. Conclusions

The investigation conducted on the possible correlations between prenatal androgen levels, self-efficacy and psychological variables in this sample of young college students made it possible to emphasize the role played by certain innate biological predispositions, in this case represented by prenatal androgen exposure levels, on the adaptive behaviors of each of us and to reiterate the importance of personal affective states in everyday life. Indeed, our study found that the 2D:4D ratio shows a negative correlation with levels of perceived self-efficacy, and in women especially, it also correlates positively with levels of state and trait anxiety. In addition, the results confirmed the adaptive role of using positive emotional regulation mechanisms.

In conclusion, our study confirmed the hypothesis of a correlation between perceived self-efficacy and prenatal androgen levels (2D:4D ratio), emphasizing the importance of innate biological factors in the architecture of personality and psychological attitudes. Moreover, our results showed that these two variables have close links with affective states, particularly anxiety, suggesting that perceptions of one’s self-efficacy and level of emotional control seem to be somewhat influenced by an innate predisposition related to prenatal androgen values to which individuals were exposed during uterine life.

## 6. Future Research

In our previous studies, we have directed our attention to various samples, such as medical students [35], subjects practicing specific sports such as skydiving [18] and cavers [51], so for this study, we decided to focus on young college students practicing sports.

In our future research, we intend to increase the sample size and extend this study to different groups of subjects, such as those with emotional–relational disorders, to investigate whether prenatal androgen exposure levels may play an important role in the etiopathogenesis and clinical management of such disorders.

## Figures and Tables

**Table 1:** The descriptive characteristics of the sample ((**A**) female sample, (**B**) male sample) aged.

A			B		
**N.**	**Age**	**D2:D4**	**N.**	**Age**	**D2:D4**
1	30	0.92	1	27	0.92
2	26	0.95	2	25	0.93
3	26	0.96	3	28	0.93
4	26	0.96	4	25	0.95
5	25	0.96	5	28	0.95
6	28	0.96	6	27	0.96
7	28	0.96	7	27	0.96
8	22	0.97	8	24	0.97
9	26	0.97	9	25	0.97
10	20	0.97	10	27	0.98
11	20	0.97	11	25	0.99
12	27	0.97	12	28	0.99
13	25	0.98	13	27	1.00
14	26	0.99	14	26	1.00
15	29	0.99	15	27	1.00
16	27	1.00	16	24	1.00
17	24	1.00	17	26	1.00
18	24	1.00	18	23	1.01
19	27	1.00	19	31	1.01
20	21	1.01	20	27	1.03
21	27	1.01	21	25	1.03
22	23	1.02	22	24	1.03
23	25	1.03	23	28	1.04
24	29	1.03			
25	31	1.03			
26	28	1.04			
27	31	1.04			
28	23	1.05			
29	21	1.05			
30	22	1.05			
31	25	1.05			
32	25	1.08			

**Table 2:** ERQ, mean and standard deviation of total, female and male samples.

	Cognitive Reappraisal		Emotional Suppression	
	M	DS	M	DS
Total	4.7341	1.0361	3.3348	1.3933
Female	4.8956	1.1155	3.0625	1.5319
Male	4.5187	0.8973	3.6979	1.1131

**Table 3:** STAI-Y, mean and standard deviation of total, female and male samples.

	State		Trait	
	M	DS	M	DS
Total	52.82	10.74	54.23	11.56
Female	52.09	10.90	51.63	10.65
Male	53.79	10.68	57.71	12.03

**Table 4:** Mean and standard deviation of factors of POMS: Tension (T), Depression (D), Aggressiveness (A), Vigor/Activity (V), Fatigue (F), Confusion (C) and Total Mood Disturbance (TMD).

	T	D	A	V	F	C	TMD
Total							
M	52.21	52.16	55.41	53.14	56.88	56.32	221.11
DS	9.13	10.70	12.23	10.64	12.21	10.14	52.49
Female							
M	51.48	50.69	53.88	51.78	56.94	54.81	218.62
DS	9.44	8.69	10.05	11.76	12.94	10.14	49.06
Male							
M	50.72	51.53	54.58	52.74	54.62	55.41	216.33
DS	8.95	12.84	14.63	8.84	11.44	9.99	57.55

**Table 5:** Factors of BFQ-2. Mean and standard deviations of total, female, and male samples.

	Energy	Agreeab.	Conscient.	Emot. Stab.	Openness	Lie
Total						
M	54.50	53.73	54.00	47.05	52.96	46.00
DS	8.80	10.62	9.86	11.24	7.62	7.94
Female						
M	54.09	54.31	53.31	46.81	50.56	45.71
DS	9.05	10.56	10.69	10.01	8.06	8.47
Male						
M	55.04	52.96	54.91	47.38	56.17	46.39
DS	8.61	10.89	8.78	12.92	5.70	7.32

## Data Availability

The datasets generated for this study are available on request to the corresponding author.
